# The Many Faces of Metabolic Dysfunction-Associated Fatty Liver Disease Treatment: From the Mediterranean Diet to Fecal Microbiota Transplantation

**DOI:** 10.3390/medicina60040563

**Published:** 2024-03-29

**Authors:** Ludovico Abenavoli, Maria Luisa Gambardella, Giuseppe Guido Maria Scarlata, Ilaria Lenci, Leonardo Baiocchi, Francesco Luzza

**Affiliations:** 1 Department of Health Sciences, University “Magna Graecia”, Viale Europa, 88100 Catanzaro, Italy; marialuisa.gambardella@studenti.unicz.it (M.L.G.); giuseppeguidomaria.scarlata@unicz.it (G.G.M.S.); luzza@unicz.it (F.L.); 2 Hepatology and Liver Transplant Unit, University of Tor Vergata, Via Montpellier, 00133 Rome, Italy; ilaria.lenci@uniroma2.it (I.L.); baiocchi@uniroma2.it (L.B.)

**Keywords:** steatosis, probiotics, gut–liver axis, leaky gut, Mediterranean diet

## Abstract

The gastrointestinal tract is inhabited by the gut microbiota. The main phyla are Firmicutes and Bacteroidetes. In non-alcoholic fatty liver disease, now renamed metabolic dysfunction-associated fatty liver disease (MAFLD), an alteration in Firmicutes and Bacteroidetes abundance promotes its pathogenesis and evolution into non-alcoholic steatohepatitis, liver cirrhosis, and hepatocellular carcinoma. For this reason, early treatment is necessary to counteract its progression. The aim of the present narrative review is to evaluate the different therapeutic approaches to MAFLD. The most important treatment for MAFLD is lifestyle changes. In this regard, the Mediterranean diet could be considered the gold standard in the prevention and treatment of MAFLD. In contrast, a Western diet should be discouraged. Probiotics and fecal microbiota transplantation seem to be valid, safe, and effective alternatives for MAFLD treatment. However, more studies with a longer follow-up and with a larger cohort of patients are needed to underline the more effective approaches to contrasting MAFLD.

## 1. Introduction

**Figure 1:**
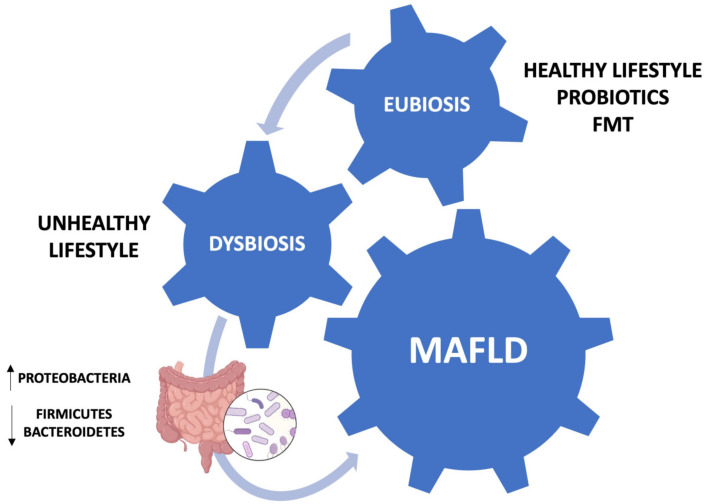
Schematic representation of the involvement of the gut–liver axis in MAFLD pathogenesis.

The nomenclature non-alcoholic fatty liver disease (NAFLD), coined in 1980, indicates the presence of fatty liver disease in the absence of other chronic liver diseases or alcohol consumption of more than 140 g/week for women and 210 g/week for men. However, due to the dysmetabolic comorbidities that commonly affect NAFLD patients, it was recently renamed to metabolic dysfunction-associated fatty liver disease (MAFLD) [1]. MAFLD is a clinical condition mainly characterized by the accumulation of fat in the liver parenchyma (>5% of hepatocytes). The pathological spectrum ranges from simple fatty liver to non-alcoholic steatohepatitis (NASH), liver cirrhosis, and hepatocellular carcinoma (HCC). More advanced stages of the disease are associated with higher mortality, but all stages of MAFLD can significantly increase the risk of cardiovascular disease. MAFLD is a common cause of chronic liver disease worldwide. The histopathological sign of MAFLD is represented by hepatic steatosis, characterized by the accumulation of lipid droplets in hepatocytes. Signs of cell damage such as swelling, apoptotic changes, and Mallory–Denk bodies are also typical, while portal and lobular inflammatory infiltrates with or without fibrosis are more characteristic of the NASH stage. The global incidence of MAFLD is 46 cases per 1000 individuals [2]. In recent years, the global prevalence of the disease has been steadily increasing, from 25.3% in 1990–2006 to 38% in 2016–2019. In addition, the prevalence in men is higher than in women (40% and 26%, respectively). However, these data are affected by some bias, including the different lifestyle and the body mass index (BMI) of the individuals being investigated [2,3]. Data from South America are scarce. In Brazil, Chile, Mexico, and Colombia the prevalence in normal-weight individuals was 35.2%, 23%, 17%, and 26.6%, respectively [4]. The pathophysiological mechanisms underlying MAFLD are usually explained by the two-hit hypothesis, in which two damaging events occurring in sequence compromise the function and structure of the liver parenchyma, leading to the accumulation of fatty acids in the liver, and, subsequently, the progressive appearance of oxidative stress and hepatocyte damage [5]. This classic scheme is considered obsolete and has been replaced by the concept of multiple hits acting in parallel, which takes into consideration insulin resistance, oxidative stress, genetic and epigenetic factors, the gut microbiota, and environmental factors [6]. The diagnosis of MAFLD is based on the presence of a fatty liver detected by ultrasonography in the absence of the other causes (virus, alcohol, drugs), and the presence of dysmetabolic comorbidities such as being overweight or obese, hypertension, or type 2 diabetes mellitus. In fact, the histological evaluation of the liver is not required for the diagnosis of MAFLD [7]. Proper management of these patients is necessary to prevent some liver complications, such as NASH, liver cirrhosis, and HCC. There is considerable evidence of a link between MAFLD, dysbiosis, and lifestyle; namely, that the synergy between the Mediterranean diet (MD), physical activity, and gut eubiosis promotes liver health. The MD emphasizes the consumption of fruits, vegetables, whole grains, legumes, nuts, seeds, olive oil, and a moderate intake of fish, poultry, and red wine. This dietary pattern is rich in fiber, antioxidants, and healthy fats, while being low in processed foods, sugars, and saturated fats. A strong adherence to the MD can significantly improve liver health and reduce the risk of fatty liver disease progression [8]. The diet’s anti-inflammatory properties, coupled with its ability to regulate lipid metabolism and insulin sensitivity, contribute to its protective effects on the liver. Additionally, the high content of polyphenols and omega-3 fatty acids found in the MD may help mitigate oxidative stress and inflammation in the liver, thereby reducing hepatic fat accumulation [9]. In this context, probiotics and fecal microbiota transplantation (FMT) have become promising treatments, based on the pivotal role of the “gut–liver axis” in the progression of MAFLD (Figure 1). Indeed, FMT can modulate the composition and function of the gut microbiota, leading to improvements in liver function and metabolic parameters. By restoring microbial diversity and promoting the growth of beneficial bacteria, a FMT may help to alleviate hepatic inflammation, insulin resistance, and lipid accumulation in the liver [10]. The aim of the present narrative review is to evaluate the different therapeutic approaches to MAFLD.

## 2. Materials and Methods

We performed a search on PubMed and Medline for original articles, reviews, meta-analyses, and editorials using the following keywords, their acronyms, and their associations: metabolic dysfunction-associated fatty liver disease, MAFLD, non-alcoholic fatty liver disease, NAFLD, fecal microbiota transplantation, FMT, Mediterranean diet, probiotics, gut microbiota. The last Medline search was dated 31 January 2024. Specifically, after the removal of duplicates, we had retrieved 422 contributions. We successively excluded 210 contributions because of the type of the paper (non-English publication, abstracts). Finally, 151 further contributions were excluded because they did not address the searched topic. Out of 61 contributions, we included 27 reviews; 11 meta-analyses; 6 editorials; and 17 original articles (namely, retrospective, perspective, multicentric, and international studies).

## 3. Gut Dysbiosis and MAFLD

The gastrointestinal tract is inhabited by the gut microbiota, a heterogeneous ecosystem of 10^14^ bacteria. The main phyla are Firmicutes and Bacteroidetes, followed by Actinobacteria, Cyanobacteria, Fusobacteria, Proteobacteria, and Verrucomicrobia [11]. Other components are fungi, archaea, phages, and viruses [12]. The microbiota begins to colonize the host at the moment of birth, although the paradigm of uterine sterility has recently been challenged. During and after birth, the neonatal gut is colonized by a variety of microbes. This process is conditioned by several factors: mode of birth, type of breastfeeding, hygienic conditions, exposure to antibiotic treatments. Usually, the gut microbial population takes on the configuration of an adult microflora during the first five years of life, even though it represents an ecosystem with dynamic evolution. With a population of over 100 trillion microorganisms, the gastrointestinal tract is one of the most complex ecosystems found in nature. The gut microbiota is defined as a superorganism that is essential for host health and performs various functions such as immune homeostasis, which is essential in counteracting colonization by pathogenic bacteria and in maintaining the integrity of the intestinal barrier. In addition, it supports the health of the host by promoting the absorption of nutrients by providing enzymatic pathways that the host lacks. It also promotes the production of vitamins K and B, and short-chain fatty acids (SCFAs) [13]. The interaction between the gut microbiota, the immune system, and the liver is defined as “gut–liver axis” [14]. Gut dysbiosis is an alteration in the structure and function of the gut microbiota, characterized by a decrease in “good” bacteria abundance and an increase in “bad” bacteria abundance, or a reduction in bacterial diversity. For this reason, it plays a central role in the pathogenesis of MAFLD, as the gut microbiota shows a reduced diversity at the phylum and family level [15]. In patients with MAFLD, an increase in Proteobacteria at the phylum level, Enterobacteriaceae at the family level, and *Escherichia*, *Dorea*, *Peptoniphilus* at the genus level was observed, when compared with healthy individuals. At the same time, a decrease was found in Rikenellaceae and Ruminococcaceae at the family level and in *Anaerosporobacter*, *Coprococcus*, *Eubacterium*, *Faecalibacterium* and *Prevotella* at the genus level [16]. In a cross-sectional study, the gut microbiota of MAFLD patients was analyzed using next-generation sequencing. As reported by the Authors, the Firmicutes/Bacteroidetes ratio was positively correlated with liver steatosis in the obese group [17]. Gut dysbiosis increases the production of SCFAs, leading to increased fat accumulation in the liver. SCFAs bind to G protein-coupled receptors 43 and 41, which are also expressed in adipocytes, inhibiting lipolysis and adipocyte differentiation. Furthermore, elevated levels of SCFAs stimulate the expression of carbohydrate response element binding protein (ChREBP). Monosaccharides from microbial fermentation activate hepatic ChREBP and consequently increase the levels of proteins involved in hepatic lipogenesis [18]. In addition, very-low-density lipoprotein synthesis is reduced with a consequent decrease in hepatic lipid export. Moreover, a gut imbalance promotes hepatic inflammation by increasing intestinal permeability, known as “leaky gut” [19]. The translocation of bacteria and pathogen-associated molecular pattern molecules stimulates an inflammatory response in the liver and, subsequently, steatosis [20]. In summary, in MAFLD there is a disequilibrium in the Firmicutes/Bacteroidetes ratio, and this promotes its pathogenesis and the development of NASH, liver cirrhosis, and HCC [20].

## 4. Dietary Regimens in MAFLD

MAFLD is considered to be the hepatic manifestation of metabolic syndrome, which can be exacerbated by a high-calorie diet in genetically predisposed individuals [21]. Obesity plays a central role in the development of MAFLD: patients are mainly obese or overweight, with only a small number consisting of lean subjects [22]. Two of the main dietary approaches are the MD and the Western diet (WD). The MD is a diet characterized by low levels of saturated fat and high levels of vegetable oils. Furthermore, it contains several natural compounds with antioxidant, anti-inflammatory, antihypertensive, lipid-lowering, anti-diabetic, and anti-obesity effects [23]. For example, extra virgin olive oil with a high oleocanthal content is associated with a reduction in BMI, transaminases, and cytokines levels [22]. Tomatoes have the main component lycopene (LYC) which reduces serum and hepatic fat levels, but the mechanism of this is still unclear. In addition, LYC induces the expression of cellular antioxidant enzymes and reduces the activity of reactive oxygen species-producing enzymes [23]. A prospective cohort study showed that the MD improved patients’ anthropometric parameters and lipid profile, and reduced hepatic steatosis and liver stiffness. In addition, it underlined that the combination of antioxidant complex and diet improved insulin resistance, hepatic steatosis, and liver stiffness, when compared with a control diet [24]. Another study evaluated the clinical efficacy of the MD in MAFLD patients. At the end of the treatment, BMI, waist circumference, waist-to-hip ratio, aspartate amino transferase (AST), alanine amino transferase (ALT), gamma-glutamyl transferase (GGT), high-density lipoproteins (HDL), low-density lipoproteins (LDL), triglycerides (TG), serum glucose, total-cholesterol/HDL ratio, LDL/HDL ratio, TG/HDL ratio, homeostatic model assessment-insulin resistance (HOMA-IR), the fatty liver index (FLI), the Kotronen index, and the fatty liver score all showed a significant improvements (*p* < 0.01) [25]. On the contrary, the WD is a dietary regimen rich in protein, fat, and refined sugars characterized by overeating, frequent snacking, and a prolonged postprandial state. In a study performed by Kübeck et al., the gut microbiota of high-fat diet-induced obese mice was transferred to germ-free mice. This transfer caused metabolic syndrome with an alteration to the epithelial barrier [26]. This dietary approach has been linked to the promotion of dysbiosis and MAFLD [27]. In this regard, a large prospective cohort study evaluated the effects of the WD diet and a Prudent diet in 3527 patients with MAFLD, 1643 with liver cirrhosis, and 669 patients with liver cancer. The dietary pattern was assessed with a food questionnaire. The Authors underlined the correlation between a WD and an increased risk of chronic liver diseases, while the Prudent diet was associated with a lower risk of liver cirrhosis [28]. These data showed the effect of lifestyle in the progression and prevention of MAFLD. In fact, the most important treatment for MAFLD has been shown to be lifestyle modification [29]. The MD could be considered the gold standard in the prevention and treatment of MAFLD, and for this reason, a strict adherence to the traditional MD can help MAFLD patients in achieving a healthy state. On the contrary, the WD should be discouraged [30]. Table 1 summarizes the studies about the use of different dietary regimens in MAFLD patients.

**Figure 2:**
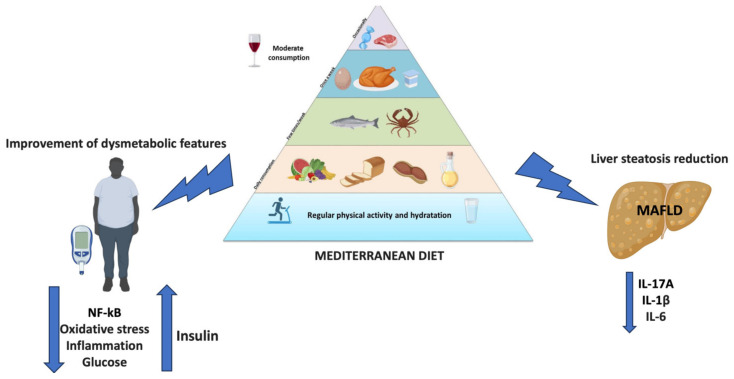
Beneficial effects of the MD in MAFLD.

Overall, the MD, with its antioxidant and anti-inflammatory food items, helps to reduce liver steatosis and improve dysmetabolic comorbidities, making it a valid therapeutic option (Figure 2).

## 5. Use of Probiotics in MAFLD

Probiotics are defined by the Food and Agriculture Organization of the United Nations and the World Health Organization as “live microorganisms that, when administered in sufficient quantities, confer a health benefit on the host”. Probiotics manipulate the gut microbiota to improve its homeostasis [31]. In fact, recent evidence showed their efficacy in antibiotic-associated diarrhea, inflammatory bowel diseases (IBDs), and colorectal cancer [32]. The use of probiotics has been associated with beneficial effects in MAFLD in several studies [33]. In a double-blind, single-center clinical trial MAFLD patients were randomly chosen to receive Symbiter or a placebo. For 8 weeks, the Symbiter group received a concentrated biomass of 14 probiotic bacteria genera such as *Bifidobacterium*, *Lactobacillus*, *Lactococcus*, *Propionibacterium* every day, while the placebo group received a placebo every day. The research team evaluated the changes in the FLI and liver stiffness, which were measured by shear wave elastography. At the end of the administration, both the placebo and probiotics were well tolerated. In the probiotic group, the FLI significantly decreased compared to the placebo group. In fact, it decreased from 84.33 ± 2.23 to 78.73 ± 2.58 in the probiotic group (*p* < 0.001), whereas it did not change in the placebo group. However, there was no significant difference in liver stiffness [34]. Another randomized controlled trial analyzed the effect of the administration of *Lactobacillus acidophilus*, *Lactobacillus rhamnosus*, *Lactobacillus paracasei*, *Pediococcus pentosaceus*, *Bifidobacterium lactis*, and *Bifidobacterium breve* in obese MAFLD patients for 12 weeks. At the end of the study, the intrahepatic fat fraction decreased from 16.3 ± 15% to 14.1 ± 7.7% in the probiotics group (*p* = 0.032), while it did not change in the placebo group. In addition, the reduction in TG levels was also more significant in the probiotic group than in the placebo group [35]. A pilot study analyzed the effect of a dosage of 500 million *Lactobacillus bulgaricus* and *Streptococcus thermophilus* in MAFLD patients. For three months, group 1 was treated with probiotics administered daily and group 2 received a placebo. After treatment, in group 1, the ALT, AST, and GGT levels decreased from 67.7 ± 25.1 to 60.4 ± 30.4 UI/L (*p* < 0.05), from 41.3 ± 15.5 to 35.6 ± 10.4 UI/L (*p* < 0.05), and from 118.2 ± 63.1 to 107.7 ± 60.8 UI/L (*p* < 0.05), respectively. In contrast, in group 2 these parameters remained unchanged. In both groups no modifications to anthropometric parameters or cardiovascular risk factors took place [36]. Mohamad et al. showed that the use of six different *Lactobacillus* and *Bifidobacterium* species improved intestinal permeability with a reduction in fat absorption [37]. Clinical trials on the use of probiotics in patients with MAFLD are summarized in Table 2. Furthermore, the beneficial effect of probiotics has also been observed with pre-clinical and clinical studies in NASH models. A study performed in obese mice with NASH showed a reduction in histological liver steatosis and transaminase levels after the administration of VSL#3 (containing *Bifidobacterium*, *Lactobacillus*, and *Streptococcus* genera) [38]. In an open-label trial on patients with NASH, one group received a probiotic cocktail (containing *Lactobacillus*, *Bifidobacterium,* and *Streptococcus* genera) for 12 weeks. These patients showed a significant (>20%) reduction in serum ALT, liver stiffness, BMI, and serum cholesterol levels when compared with the control group [39]. In summary, the use of *Bifidobacterium* and *Lactobacillus* as probiotics improves gut dysbiosis, which is often associated with a WD [12]. The restoration of gut eubiosis seems to grant a beneficial effect in MAFLD and NASH patients. However, new studies with a larger sample and a longer follow-up are necessary to confirm their use in clinical practice.

## 6. FMT in MAFLD Patients

FMT consists of the transfer of stool from a healthy donor to a patient with gut dysbiosis [40]. The therapeutic benefit of FMT is determined by its capacity to restore the gut microflora composition [41]. FMT can be administered by enema, into the upper gastrointestinal tract, by colonoscopy, or using oral capsules [42]. The requirements for FMT donors are age < 60 years and healthy status, while exclusion criteria are risk of infectious disease, gastrointestinal comorbidities, and factors that may affect the composition of the gut microbiota: systemic auto-inflammatory disease, atopic disease, metabolic syndrome, obesity, moderate/severe malnutrition, chronic pain syndromes, pregnancy, previous or planned gastrointestinal surgery, or a history of cancer [43]. FMT showed a high success rate in treating gastrointestinal infectious diseases, particularly *Clostridium difficile* infection [44]. In addition, recent studies have shown that FMT is also effective in IBD patients [45]. However, it is less effective in IBD patients than in those patients colonized by *Clostridium difficile*. Therefore, the response could be due to the differences between the recipient’s and donor’s gut microbiota composition [46]. Autologous FMT is based on the use of collected feces to restore gut microbial communities after perturbations. This approach is a better alternative to traditional FMT (defined as allogeneic FMT) [47]. As previously reported, probiotics improve intestinal permeability and have beneficial effects in MAFLD patients [33]. However, there are no studies that have evaluated the correct dose and strain of probiotics and their adverse effects in MAFLD patients. Therefore, the use of live commensals from a healthy gut may be safer and more effective than probiotics. Few studies have evaluated FMT efficacy in MAFLD patients. Xue et al. divided MAFLD patients into an FMT group, non-FMT group, and healthy controls. The non-FMT group received oral probiotics (*Bifidobacterium* and *Lactobacillus acidophilus*, respectively), while the FMT group received 200 mL of bacterial cocktail from healthy donors for 3 days. This randomized controlled trial showed that FMT decreased the fat accumulation in the liver by improving the gut microbiota dysbiosis and the fatty liver disease. However, there were no statistical differences between the FMT and non-FMT groups in terms of liver function, hepatic fat accumulation, and blood lipid levels. In addition, this study showed that FMT had a better effect in lean MAFLD patients than in obese MAFLD patients [48]. Another study compared the two different types of FMT in MAFLD patients. As reported by the Authors, allogeneic FMT improved intestinal permeability better than autologous FMT. However, there were no significant statistical differences in insulin resistance or hepatic proton density fat fraction between autologous and allogeneic FMT [49]. Witjes et al. evaluated the effects of allogeneic FMT from a lean vegan donor via nasoduodenal tube in MAFLD/NASH patients. A liver biopsy was performed and the markers of steatohepatitis were assessed at baseline and after 24 weeks. At the end of the study, they showed that allogeneic FMT improved patients’ necro-inflammatory histology and bio-humoral liver profile [50]. Finally, a recent review underlined that FMT had good preclinical and clinical results in MAFLD patients [51]. Clinical trials on the application of FMT in patients with MAFLD are summarized in Table 3.

## 7. Discussion

NAFLD is a common cause of chronic liver disease worldwide. Due to several dysmetabolic comorbidities found in patients with fatty liver disease, its nomenclature has been recently changed to MAFLD. The correct management of MAFLD-patients and the use of novel potential biomarkers are crucial in preventing MAFLD-related liver complications, such as NASH, liver cirrhosis, and HCC [52]. Currently, the only therapy recognized by the scientific community involves a lifestyle change supported by a balanced diet. For this reason, several clinical studies have been performed in support of the use of the MD in the treatment of MAFLD. These studies have revealed how it acts both by reducing metabolic comorbidities, such as obesity and insulin resistance, and by reducing liver steatosis, liver biomarker levels, and related scores [24,25]. Conversely, the therapeutic approach based on the WD significantly increased the risk of chronic liver diseases [28]. However, although the MD has been proven to be effective in counteracting MAFLD, the association of additional substances with antioxidant action or regular physical exercise is often necessary to improve its efficacy. The latter has been the subject of a recent meta-analysis that confirmed how the combined use of MD and physical activity reduces metabolic risk factors, preventing the development of metabolic syndrome. However, the studies under investigation were very heterogeneous in terms of sample size and type of intervention [53]. In the case of both metabolic syndrome and MAFLD, new randomized controlled trials with a larger number of patients and longer follow-up are needed to validate the therapeutic role of MD in dysmetabolic etiology pathologies. Another possible therapeutic alternative is the use of probiotics with the aim of restoring intestinal eubiosis, which, if altered, contributes to the pathogenesis of MAFLD. However, studies have shown contrasting effects. While there was a significant improvement in intestinal permeability, bio-humoral liver profile, and in fat absorption, when compared to the placebo group, no such improvement was found in terms of anthropometric parameters or liver stiffness. Moreover, the studies had a very variable observation period (ranging from 8 to 24 weeks), and the exact dose of probiotics to be administered, as well as potential adverse effects, is not well defined [34,35,36,37]. Despite this, probiotics are still under investigation in pathologies involving the gut–liver axis [54,55,56]. Finally, randomized controlled trials have demonstrated that FMT is more effective than probiotic therapy in restoring gut eubiosis. However, its efficacy is mainly associated with a lean MAFLD phenotype, which can be quite limiting from a clinical perspective, as MAFLD is more strongly associated with obesity [20,48]. At the same time, the efficacy of the two transplants, autologous and allogeneic, has been compared: the latter showed significant improvements in intestinal permeability, inflammation, and the bio-humoral liver profile, while insulin resistance and hepatic proton density fat fraction did not show statistically significant differences [49,50]. Although there are still few studies regarding MAFLD, FMT has been effective in treating *Clostridium difficile* infection and IBD [44,45]. In the first case, FMT has been shown to be more effective than conventional therapy in eradicating *Clostridium difficile* infection, especially recurrences [57]. Similarly, in patients with IBD, FMT has led to significant clinical remission compared to the placebo group, with few mild side effects that resolved rapidly [58]. Further studies on a larger number of patients and on side effects are needed to support the use of FMT as a therapeutic option for MAFLD.

## 8. Conclusions and Future Perspectives

Treating MAFLD poses a challenge for public health. The only therapy currently recognized involves a lifestyle change through a multidisciplinary approach that includes various specialists. Among the new valid therapeutic options, the MD is probably the safest and least invasive approach, and it has led to significant improvements in the outcomes of patients with MAFLD. At the same time, FMT has proven to be more effective than probiotics in restoring intestinal eubiosis, despite being a potentially invasive method and being most effective in lean patients. Additionally, recent studies have shown few, generally well-tolerated, side effects. Conversely, probiotics have been effective in obese MAFLD patients. Overall, all of these approaches have been shown to be promising for the treatment of patients with MAFLD, although more studies with a longer follow-up and a larger cohort of patients are needed to evaluate their potential application in clinical practice. Among these, FMT is one of the most studied, especially in liver diseases and IBD [59,60]. In addition to performing new studies on the aforementioned approaches, medicine should move towards researching new therapies in order to attempt a targeted and personalized approach for the patient. Among these, microRNAs (miRNAs) are being investigated. In this regard, a recent study has shown that the overexpression of miR-129-5p interferes with the pathways of various target genes and has potential protective and therapeutic effects for many diseases involving the brain–gut–liver axis [61]. However, further analyses are needed to validate their use in the diagnosis and therapy of MAFLD.

## Figures and Tables

**Table 1:** Summary of studies about the use of different dietary regimens in MAFLD patients.

Study Design	Study Groups	Intervention	Outcomes
Randomized controlled trial [24]	Overweight-MAFLD group (*n* = 50)	Moderately hypocaloric MD or MD diet and antioxidant supplementation or no treatment for six months	Significant improvement in anthropometric parameters, lipid profile, liver steatosis, and liver stiffness in group treated with MD diet and antioxidant supplementation
Uncontrolled trial [25]	MAFLD group (*n* = 46)	MD and moderate physical activity for 6 months	Significant improvement of BMI, waist circumference, waist-to-hip ratio, AST, ALT, GGT, HDL, LDL, TG, serum glucose, total-cholesterol/HDL ratio, LDL/HDL ratio, TG/HDL ratio, HOMA-IR, FLI, Kotronen index, and fatty liver score
Prospective cohort study [28]	MAFLD group (*n* = 3527) vs. liver cirrhosis group (*n* = 1643) vs. liver cancer group (*n* = 669)	WD or Prudent diet	WD was significantly associated with increased risk of chronic liver diseases; Prudent diet was significantly associated with a lower risk of liver cirrhosis

Abbreviations: MAFLD, metabolic dysfunction-associated fatty liver disease; MD, Mediterranean diet; BMI, body mass index; AST, aspartate amino transferase; ALT, alanine amino transferase; GGT, gamma-glutamyl transferase; HDL, high-density lipoproteins, LDL, low-density lipoproteins; TG, triglycerides; HOMA-IR, homeostatic model assessment-insulin resistance; FLI, fatty liver index; WD, Western diet.

**Table 2:** Summary of clinical trials about the use of probiotics in MAFLD patients.

Study Design	Study Groups	Intervention	Outcomes
Randomized controlled trial [34]	MAFLD group (*n* = 59)	Administration of Symbiter or placebo for 8 weeks	FLI significantly decreased in probiotic group. Probiotics significantly reduced the level of serum AST and GGT No significant difference in liver stiffness among groups
Randomized controlled trial [35]	Obese-MAFLD group (*n* = 69)	Administration of probiotics or placebo for 12 weeks	Significant decrease in the intrahepatic fat fraction and in TG levels in the probiotics group
Randomized controlled trial [36]	MAFLD group (*n* = 28)	One tablet per day with 500 million *Lactobacillus bulgaricus* and *Streptococcus thermophilus* or with one placebo tablet (120 mg of starch) for 3 months	ALT, AST, and GGT levels significantly decreased in the group treated with probiotics. No significant changes in anthropometric parameters
Randomized controlled trial [37]	MAFLD group (*n* = 46)	Administration of probiotics or placebo for 6 months	Significant improvement in intestinal permeability with a reduction in fat absorption after probiotics treatment

Abbreviations: MAFLD, metabolic dysfunction-associated fatty liver disease; FLI, fatty liver index; AST, aspartate amino transferase; GGT, gamma-glutamyl transferase, TG, triglycerides; ALT, alanine amino transferase.

**Table 3:** Summary of clinical trials about the application of FMT in MAFLD patients.

Study Design	Study Groups	Intervention	Outcomes
Randomized controlled trial [48]	FMT group (*n* = 47) vs. non-FMT group (*n* = 28) vs. healthy controls (*n* = 10)	Administration of probiotics in non-FMT group. Administration of 200 mL of bacterial cocktail from healthy donors for 3 days in FMT-group	Promotion of gut eubiosis after FMT Better efficacy of FMT among lean MAFLD patients than obese MAFLD patients
Randomized controlled trial [49]	Allogeneic FMT group (*n* = 15) vs. autologous FMT group (*n* = 6)	Allogeneic or autologous FMT	Allogeneic FMT significantly improved intestinal permeability better than autologous FMT. No significant statistical differences in insulin resistance and hepatic proton density fat fraction between autologous and allogeneic FMT
Randomized controlled trial [50]	Autologous FMT (*n* = 11) vs. allogeneic FMT (*n* = 10)	Allogeneic or autologous FMT	Allogeneic FMT significantly improved necro-inflammatory histology and bio-humoral liver profile

Abbreviations: FMT, fecal microbiota transplantation; MAFLD, metabolic dysfunction-associated fatty liver disease.
